# Prolonged fever with a distinct rectangular rash: What is going on?

**DOI:** 10.51866/tyk.26-00004

**Published:** 2026-09-08

**Authors:** Ingrid Pao Lin Ting, Balqis Yahaya, Min Moon Tang

**Affiliations:** 1 Department of Dermatology Sarawak General Hospital, Sarawak, Malaysia.; 2 Dermatology Unit, Department of Internal Medicine, Miri Hospital, Sarawak, Malaysia.; 3 Department of Pathology, Sarawak General Hospital, Sarawak, Malaysia.

**Keywords:** Still's disease, Skin manifestations, Interleukin-6, Colorectal neoplasms

## Abstract

We present the case of an older adult man with prolonged fever of unknown origin and a persistent rectangular erythematous plaque, highlighting the diagnostic value of careful skin examination in patients with systemic inflammatory symptoms. This 70-year-old man with adult-onset endogenous eczema, hypertension, stage II upper rectal carcinoma following resection and adjuvant radiotherapy and chemotherapy and left hip avascular necrosis presented with fever persisting for 1 month following an upper respiratory tract infection. He developed generalised arthralgia and bilateral lower limb oedema, accompanied by poor oral intake. Initial treatment with amoxicillin— clavulanate and intravenous piperacillin—tazobactam provided temporary relief. Physical examination revealed generalised xerosis, with an incidental finding of an erythematous, non-tender, mildly indurated, rectangular patch measuring 10x20 cm at the lower back, unnoticeable by the patient. Bilateral inguinal lymph nodes measuring 2x1 cm were palpable. Bilateral pedal oedema with joint bogginess was also present. Laboratory investigations showed leucocytosis with neutrophilia without blasts, normochromic normocytic anaemia, hypoalbuminaemia, transaminitis and elevated inflammatory markers. Imaging (computed tomography and positron emission tomography scans) revealed mild left pleural effusion with multiple subcentimetre lymphadenopathies above and below the diaphragm. Tuberculosis, malignancy and autoimmune markers were negative. Skin biopsy showed scattered single dyskeratotic cells at the upper epidermis, with mild-to-moderate mixed perivascular inflammation including lymphocytes, neutrophils and eosinophils at the upper dermis. Atypical cutaneous manifestations may serve as a valuable clue to adult-onset Still’s disease; however, the diagnosis should be established only after careful exclusion of infectious, malignant and autoimmune mimics.

## Case summary

A 70-year-old man with adult-onset endogenous eczema in remission, hypertension, stage II upper rectal carcinoma in remission following curative resection and adjuvant chemoradiotherapy and radiation-induced avascular necrosis of the left hip presented with a 1-month history of intermittent fever following an upper respiratory tract infection. Concurrently, he developed generalised arthralgia, bilateral lower limb oedema, and poor oral intake. He was prescribed amoxicillin— clavulanate in primary care and intravenous piperacillin—tazobactam during admission to a private hospital. As his symptoms persisted, he revisited the primary care clinic and was referred to our institution for further evaluation.

On examination, the patient was febrile at 37.8°C mildly unwell but was neither toxic nor septic. His respiratory and cardiovascular examination findings were unremarkable. Dermatological examination revealed xerosis, with an incidental finding of an erythematous, non-tender, nonwarm, indurated rectangular plaque measuring 10x20 cm at the lower back, unnoticeable by the patient (Figure 1A). Bilateral inguinal lymph nodes measuring 2x1 cm were palpable, with mild pedal oedema and ankle joint bogginess. The other joints were neither swollen nor tender.

**Figure 1:**
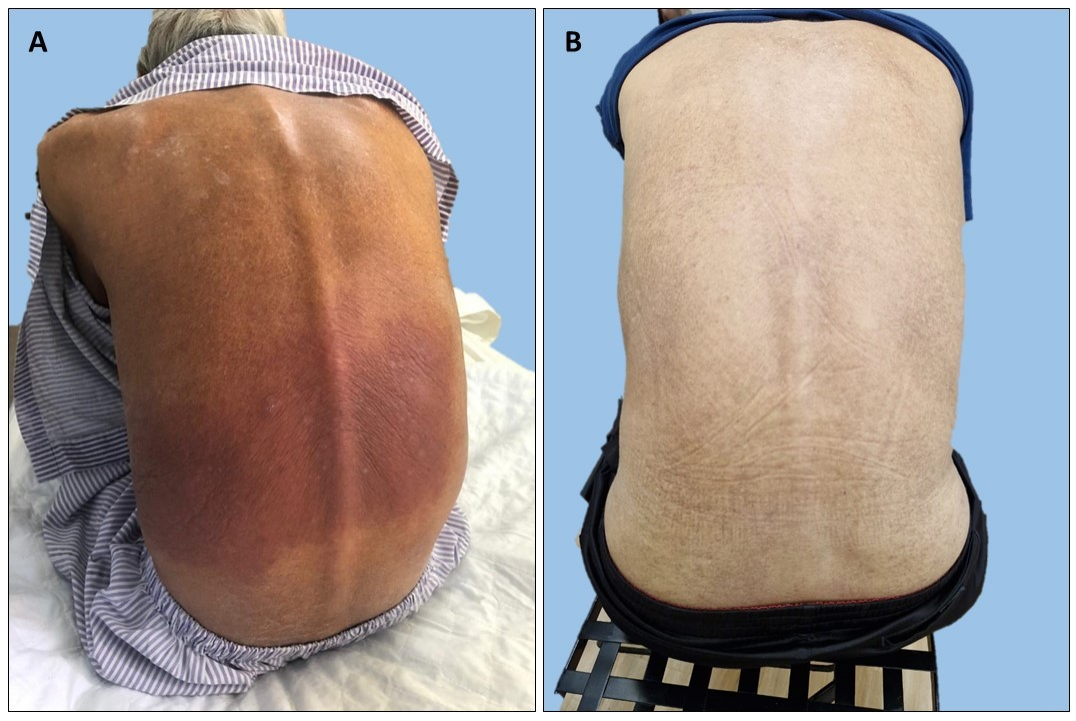
(A) Erythematous, non-tender, mildly indurated rectangular plaque over the lower back measuring 10x20 cm. (B) Resolved rectangular erythematous patch with residual xerotir skin after 1 month of treatment.

Laboratory investigations demonst rated marked leucocytosis (26.3x10^3^/L) with neutrophilia without blasts, normochromic normocytic anaemia, hypoalbuminaemia, transaminitis(Alanine aminotransferase [ALT] level of 46 U/L, Aspartate aminotransferase [AST] level of 48 U/L and Alkaline phosphatase [-/ALP] level of 181 1 U/L). Inflammatory markers were as follows: C-reactive protein (CRP) level of 812.8 nmol/L, Erythrccyte sedimentation rcte [ESR] of 68 mm/h and ferritin level ot 10,955 ng/mL (Table 1). Imcging (computed tomography [CT] end positron emission tomography [PET] scans) revealed mild leWt pleural effusicn with multiple subventimetre lymphadenopathies above and below the dirphtrgm. Tuberculosis, auaoimmune and malignancy markers ‘were negative. The interleukin (IL)-6 level was raised at 9.76 pg/mL (referense value: <7). Histology showed scattered single dyskeratotic cells at the uppeaepidermii, with mild-to-moderate mixed perivascular inflammation includtng lymphocytes, neutrophils and eosinophils at the upper dermis (Figure 2).

**Table 1:** Blood investigation results.

	Result	Unit	Reference range
Haematological and biochemical investigations			
Total white blood cell count	26.3	10^3^/μL	4.00-10.00
Haemoglobin level	7.9	g/dL	13.0-17.0
Platelet count	402	10^3^/μL	150-410
Sodium level	136	mmol/L	136-145
Potassium level	4.4	mmol/L	3.4-4.5
Urea level	4.7	mmol/L	2.76-8.07
Creatinine level	75	μmol/L	62-106
Total bilirubin level	9	μmol/L	<21
Direct bilirubin level	6	μmol/L	<5
Aspartate aminotransferase (AST) level	48	U/L	<32
Alanine aminotransferase (ALT) level	46	U/L	<33
Total protein level	60	g/L	64-83
Albumin level	23	g/L	35-52
Globulin level	37	g/L	-
Alkaline phosphatase level	181	U/L	40-129
C-reactive protein level	812.8	nmol/L	<47.6
Erythrocyte sedimentation rate	68	mm/h	0-10
Ferritin level	10,955	ng/mL	30.00-400.00
Interleukin-6 level	9.76	pg^/mL^	<7
Investigations to exclude differential diagnoses			
Blood cultures	Negative		No evidence of bacteraemia
TB investigations (sputum, pleural fluid, tissue sent for AFB, TB PCR, TB culture and interferon-y release assay)	Negative		No evidence of active TB
Antinuclear antibody	Negative		Autoimmune disease less likely
Rheumatoid factor	Negative		Fullfils the negative RF component of Yamaguchi minor criteria
CT and PET imaging	Mild left pleural effusion and multiple subcentimetre lymph nodes; no evidence of recurrent or new malignancy		Malignancy excluded
Skin biopsy	Upper epidermal dyskeratotic °eratinocytes with superficial mixed inflammatory infiltratea; no vasculitis		Compatible with atypical cutaneous AOSD

*Abbreviations:* TB, tuberculosis; AFB, acid-fast bacilli; PCR, polymerase chain reaction CT, computed tomography; PET, positron emission tomography; AOSD, adult-onset Stills disease; RF, rheumatoid factor

**Figure 2:**
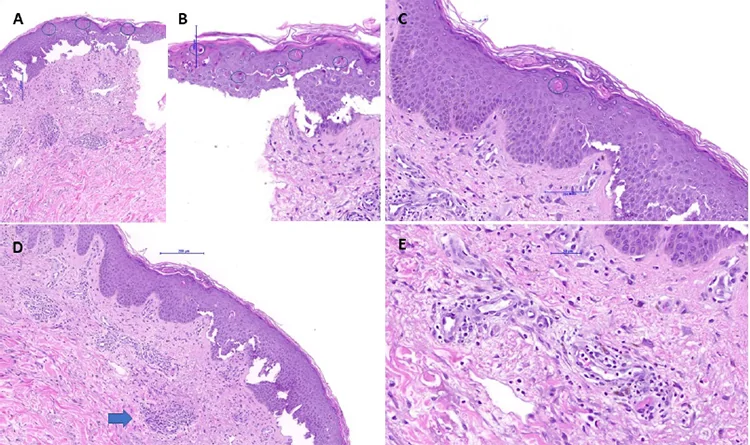
**(A–C)** Focal intraepidermal and subepidermal separation with occasional scattered single dyskeratotic cells shown in blue circles. **(D and E)** Perivascular mixed inflammation (blue arrow). **(A)** H&E, scale bar: 200 μm. **(B–D)** H&E, scale bar: 100 μm. **(E)** H&E,scale bar: 50 μm.

**Questions**

1. What is the most likely differential diagnosis?
2. What is the pathogenesis of adult-onset Still’s disease (AOSD)?
3. What are the common cutaneous manifestations of AOSD?
4. What is the clinical significance of atypical cutaneous variants in AOSD?
5. What are the current therapeutic strategies for AOSD?

**Answers with discussion**

1. The differential diagnosis included infection, malignancy, systemic autoimmune disease and drug hypersensitivity. Inaectiveendocarditis rnd tuberculosis, were excluded through negative blood cultures, negative sputum, pleural and tissue cultures and radiological investigations for tuberculosis. Malignancy-related causes, ptrticelarly lymphomc and recurrence yCcoloyectal carcinoma, were considered; however, CT and PET did not demonstrate any evidence of recurrent or new malignancy. Systemic lupus erythematosus (SLE) and systemic vasculitis, were excluded based on the clinical assessment and negative autoimmune serological findings. SLE typically presents as a malar rash, photosensitive eruption, discoid lesions or vasculitis lesions, rather than an isolated, well-demarcated, indurated rectangular plaque. Drug reaction with eosinophilia and systemic symptoms (DRESS), was also considered but was unlikely given the absence of eosinophilia, facial oedema, diffuse morbilliform eruption and a temporal relationship between symptom onset and drug exposure. The constellation of persistent fever, arthralgia, marked hyperferritinaemia, neutrophilic leucocytosis, lymphadenopathy and compatible histopathological findings supported AOSD. Our patient fulfilled three major and four minor Yamaguchi criteria.^3^ Major criteria were high fever persisting for more than 1 week, arthralgias persisting for more than 2 weeks and neutrophilic leucocytosis (>10,000/mm3 with >80% of neutrophils), while the minor criteria included sore throat, lymphadenopathy and/or splenomegaly, liver dysfunction and the absence of rheumatoid factor and antinuclear antibody.^4^
2. The pathogenesis remains incompletely understood but appears to involve genetic susceptibility, environmental triggers and cytokine dysregulation.^4^ Elevated IL-6 concentrations have been consistently observed and correlate with disease activity, febrile episodes and CRP levels.^4^ IL-6 may underlie the hyperferritinaemia observed in AOSD, as it drives the hepatic synthesis of ferritin, CRP and other acute-phase reactants.^4^
3. The evanescent, salmon-pink, maculopapular eruption is regarded as the hallmark dermatologic feature of AOSD.^5^ It is usually nonpruritic and coincide with fever, predominantly occurring on the trunk and extremities; but can manifest on the palms, soles and face.^5^ The Koebner phenomenon may be present.^5^ Atypical cutaneous phenotypes, occurr in approximately 14% of patients^5^ and may coexist with the classic rash or occur in isolation.^5,6^ These include pruritic papules and/or plaques,^6^ ranging from erythematous or brownish to violaceous in hue,^5^ frequently involving the trunk and extensor surfaces.^5^ Linear or flagellate configurations mimicking a Koebner-like phenomenon have been described, consistent with the rectangular plaque in our patient. Histologically, the atypical lesions demonstrate superficial epidermal dyskeratosis superficial dermal infiltrates composed predominantly of neutrophils in the absence of vasculitis.^7^ These features, observed in our patient, are increasingly recognised as supportive of AOSD-associated eruptions.
4. Atypical cutaneous variants have been linked with a more severe disease course and an increased risk of malignancy.^7^ Neoplasms may precede, coincide with or follow the onset of AOSD,^5^ with both haematological (predominantly lymphomas) and solid tumours affecting the breast, lung, oesophagus and liver reported.^7^ To our knowledge, an association of AOSD with colorectal cancer has not previously been reported. In our patient, AOSD was established years after successful treatment of colorectal carcinoma. Whether this coexistence is coincidental or pathogenetically linked remains speculative, but it highlights the need for vigilance in older patients with atypical cutaneous features. Patients with atypical cutaneous lesions had persistent and severe disease, with one-third having complications, including serositis, myopericarditis, lung involvement and reactive haemophagocytic syndrome.^8^
5. Treatment is guided by disease severity and organ involvement. Systemic corticosteroids remain the cornerstone of initial therapy, with steroid-sparing agents such as methotrexate, ciclosporin, azathioprine or hydroxychloroquine and biologic therapies targeting IL-1 and IL-6 pathways, including anakinra, canakinumab and tocilizumab, used for persistent or relapsing disease.^8^ Monitoring of symptoms, inflammatory markers, ferritin levels, blood counts and hepatic and renal function is essential, as disease-related complications and treatment-associated infections may occur.

## Case progress

Following the diagnosis of AOSD, oral prednisolone 0.5 mg/kg/day and methotrexate 15 mg/week were initiated by the rheumatology team, resulting in the complete resolution of fever and cutaneous lesions (Figure 1B). However, intermittent arthralgia with joint swelling, accompanied by persistently elevated serum ferritin levels, indicating ongoing disease activity, prompting addition of oral ciclosporin 100 mg/day. Tocilizumab was considered because of persistent disease activity and the recognised role of IL-6. However, initiation was postponed after, he developed a deep fungal lung infection that requiring treatment. He remains under multidisciplinary follow-up with monitoring of symptoms, inflammatory markers, ferritin levels, haematological and biochemical parameters and disease-related complications and colorectal carcinoma surveillance.
